# A Rare Multilevel Spinal Epidural Abscess With Streptococcus agalactiae in an Adult With Uncontrolled Diabetes: A Case Report

**DOI:** 10.7759/cureus.42802

**Published:** 2023-08-01

**Authors:** Karanvir S Kals, Aakriti Ramayani, Radhika Hariharan, Thomas T Lee

**Affiliations:** 1 Internal Medicine Residency Program, St. John's Riverside Hospital, Yonkers, USA; 2 Infectious Disease, St. John's Riverside Hospital, Yonkers, USA; 3 Neurological Surgery, St. John's Riverside Hospital, Yonkers, USA

**Keywords:** back pain, fever, epidural infection, spinal infection, immunocompromised, diabetes mellitus, streptococcus agalactiae, spinal decompression, spinal cord compression, spinal epidural abscess

## Abstract

Spinal epidural abscess (SEA) is a rare, life-threatening infection that typically presents with fever, back pain, and neurologic deficits. Although most commonly caused by *Staphylococcus aureus*, this case reviews a rare occurrence of a multilevel SEA caused by *Streptococcus agalactiae* in a 62-year-old female with uncontrolled type II diabetes mellitus. The patient initially presented with lower back pain and was subsequently diagnosed with a SEA complicated by hyperglycemia. A prompt diagnosis with magnetic resonance imaging (MRI) revealed extensive abscess formation, leading to emergent neurosurgical intervention. *Streptococcus agalactiae *was identified as the causative organism through culture. The report emphasizes the challenges of early detection of SEA and highlights the importance of considering unusual pathogens in high-risk patients. Timely management is crucial to prevent permanent neurologic deficits and to achieve favorable outcomes.

## Introduction

Spinal epidural abscess (SEA) is a rare, life-threatening infection that typically presents with a triad of symptoms, including fever, back pain, and neurologic deficits. Risk factors that may increase the incidence of SEAs include but are not limited to, diabetes mellitus, epidural procedures, intravenous drug use, and trauma. Prompt diagnosis using computer tomography (CT) and/or MRI, as well as swift management with surgical intervention and appropriate antibiotics, are necessary for the patient’s successful recovery. Although *Staphylococcus aureus* accounts for roughly two-thirds of bacterial SEAs, various other bacterial pathogens are known to cause SEAs. *Streptococcus agalactiae* (Group B *Streptococcus*) (GBS) is a rare cause of SEA and is the causative organism in the case presented [1]. This case report describes a 62-year-old female with uncontrolled type 2 diabetes mellitus who presented to the hospital with lower back pain. She was subsequently diagnosed with SEA, caused by GBS complicated by hyperglycemia.

## Case presentation

A 62-year-old Caucasian female with a medical history of type II diabetes mellitus and hypertension presented to the emergency department after a fall. About a week prior to admission, while at work, the patient experienced discomfort while lifting heavy boxes, which immediately led to lower back pain and weakness in her legs. The intensity of the pain prompted her to visit an urgent care center on two separate occasions, where she was prescribed cyclobenzaprine 5 mg three times a day as needed for pain relief. On the day of admission, while climbing a set of stairs, she noticed increasing weakness in her legs, causing her to fall onto her knee. She reported experiencing excessive urination and increased thirst for the two days leading up to her admission. The patient lives with her brother, who mentioned that she had been displaying signs of confusion for several days preceding her hospitalization. The brother did not endorse any recent surgical procedures. In the emergency department, the patient denied experiencing fevers, chills, loss of consciousness, dizziness, lightheadedness, visual disturbances, chest pain, palpitations, shortness of breath, wheezing, coughing, abdominal pain, diarrhea, constipation, melena, hematochezia, urinary urgency, or dysuria.

The patient's abnormal vital signs were a heart rate of 100 beats per minute and a temperature of 96.5°F. The physical exam was remarkable for a dried wound on the left third digit of the foot with a necrotic tip, and tenderness was observed upon palpation of the thoracic and lumbar spine. Motor strength was 1-2+ throughout, with noted weakness in the proximal muscle area of both legs. Deep tendon reflexes (DTRs) were assessed; the patient displayed +0/4 DTRs in bilateral knee and Achilles reflexes and +1/4 DTRs in bilateral biceps and brachioradialis reflexes. There was worsening pain in the lumbosacral region when actively raising the right lower extremity.

Table 1 presents the patient's initial laboratory results.

**Table 1:** The patient's initial laboratory results

Lab	Values	Reference Range
White Blood Cells	21.5 K/mm^3^	4.0-10 K/mm^3^
Neutrophils %	84%	42.8-82.8 %
Random Glucose	421 mg/dL	74-106 mg/dL
Carbon Dioxide	18 mmol/L	21-32 mmol/L
Anion Gap	20 mmol/L	8-16 mmol/L
Hemoglobin A1c	11.5 %	<5.7 %
Beta-Hydroxybutyrate	72.8 mg/dL	0.2-2.8 mg/dL
Venous Blood pH	7.352	7.310-7.410
Urinalysis Protein	2+	Negative
Urinalysis Glucose	3+	Negative
Urinalysis Ketones	3+	Negative
Urinalysis Blood	1+	Negative
Urinalysis Nitrites	Negative	Negative
Urinalysis Leukocyte Esterase	Negative	Negative
Urinalysis Bilirubin	Negative	Negative

Urine cultures also showed positive results for both pansensitive GBS and pansensitive *Escherichia coli*. Blood cultures revealed positive results for pansensitive GBS.

**Figure 1:**
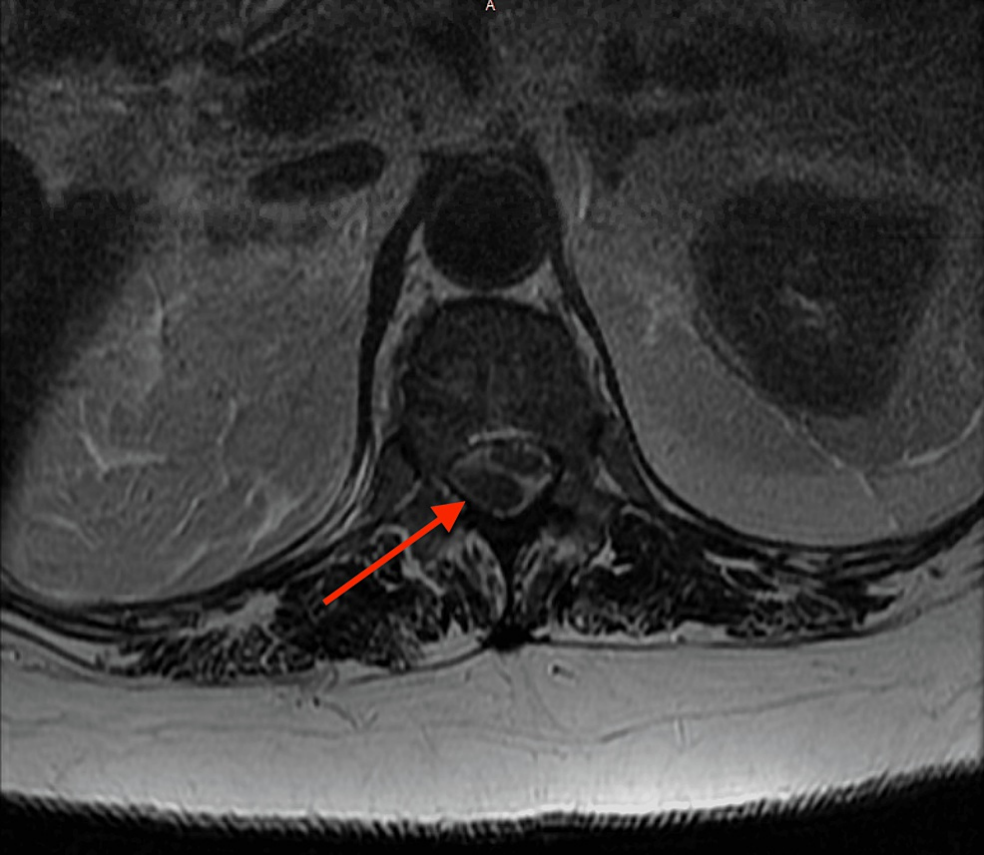
T2-weighted MRI of the thoracic spine with IV contrast; axial view

**Figure 2:**
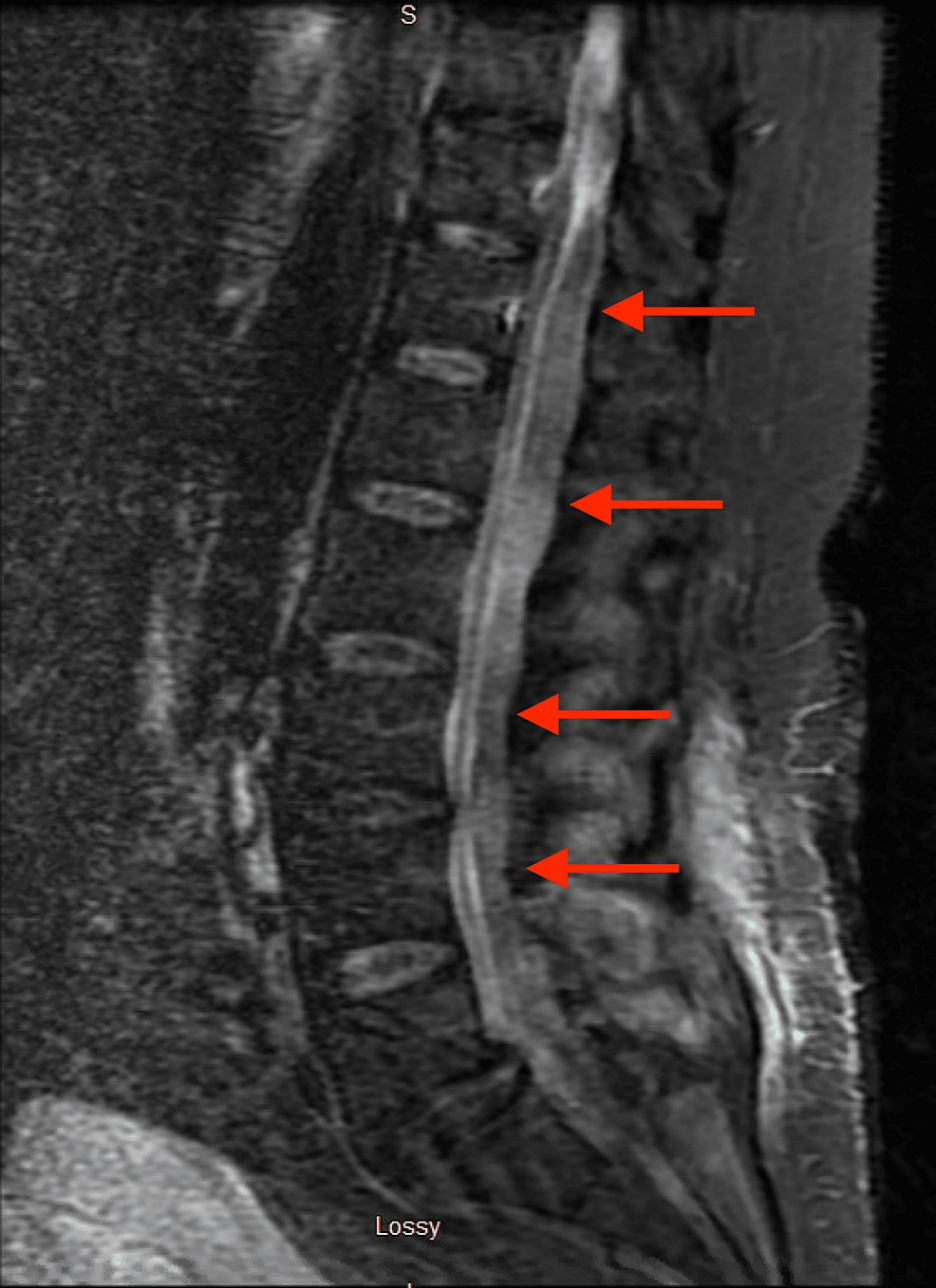
Short-T1 inversion recovery MRI of the lumbar spine without contrast; sagittal view

Multiple imaging studies were performed, including thoracic and lumbar MRIs without contrast as well as a thoracic MRI with contrast. The imaging findings indicated a contiguous, loculated extraspinal fluid extending from the T10 level through the length of the lumbar spine to the upper sacral spine at the approximate junction of the S1 and S2 segments. There was evidence of spinal cord compression at the level of the upper lumbar spine and moderate cauda equina compression at the level of the mid-lumbar spine. Additionally, there was a suspicion of right L4-L5 and L5-S1 septic facet joint arthritis and a large epidural fluid collection posterior to the thoracic cord, extending from the T10-T11 disc level to the upper lumbar region (Figures 1-2).

Prior to surgical intervention, appropriate management of hyperglycemia was carried out in the intensive care unit. The patient received initial antibiotic treatment with ceftriaxone and gentamicin for empiric coverage of endocarditis in the setting of concurrent gram-positive bacteremia and SEA. A transthoracic echocardiogram did not reveal any signs of vegetation on the heart valves. Neurosurgery promptly scheduled the patient for emergent T11-L5 laminectomies, partial T10 and S1 laminectomies, right L5-S1 facetectomy, microdissection, and debridement of the abscess. During the surgical procedure, the presence of SEA complicated by compression of the spinal cord and cauda equina was observed. Additionally, an infected and loose right L5-S1 facet joint was identified and sent for pathology, which confirmed osteomyelitis of the right L5-S1 facet bone. The SEA was cultured, and GBS was identified as the causative organism.

Following the surgery, the patient’s motor strength of the lower extremity was +4/4 distally and 4-/5 proximally, with a negative Babinski sign bilaterally and a grossly intact sensation. The patient was ultimately discharged to a skilled nursing facility to complete an eight-week course of ceftriaxone and to receive physical rehabilitation.

## Discussion

Spinal epidural abscesses (SEAs) are relatively uncommon but can have severe consequences, often posing challenges to early detection due to nonspecific symptoms. The incidence of SEA has shown an upward trend in recent decades, attributed to factors such as an aging population, increased intravenous drug abuse, and greater utilization of epidural instrumentation. A study conducted at Massachusetts General Hospital between 1947 and 1974 reported an incidence of SEA ranging from 0.2 to 1.2 per 10,000 admissions [2]. In contrast, a retrospective study covering the period from 2004 to 2014 at a large academic hospital found an incidence of 5.1 cases per 10,000 admissions [1]. These variations in incidence rates may be attributed to several factors, including the ones mentioned above, as well as differences in diagnostic and surgical approaches. Notably, the introduction of MRI in 1973 revolutionized the diagnosis and treatment of SEA, serving as the most effective diagnostic tool for its detection.

The main predisposing factors for the development of SEA include diabetes mellitus, intravenous drug abuse, chronic renal failure, trauma, and systemic immunodeficiency. The location of the abscess also plays a crucial role in treatment considerations. Spinal epidural abscesses most frequently occur at the lumbar level (48%), followed by the thoracic (31%) and cervical (21%) levels [3]. The development of SEA can be attributed to hematogenous spreading, direct spread from adjacent structures, or direct inoculation from invasive procedures [3]. *Staphylococcus aureus* is the most commonly isolated pathogen in cases of SEA. Other less frequently involved pathogens include Gram-negative bacilli (16%), *Streptococci *(9%), and anaerobic pathogens (2%) [4].

Group B *Streptococcus *is not commonly found in spinal epidural abscesses. Typically, GBS is present in the gastrointestinal or genitourinary tract. When the bacterium invades the non-pregnant adult population, GBS is usually associated with bloodstream infections, lung infections, skin infections, or bone infections [5]. In a meta-analysis involving 101 cases of SEAs, only one out of the 101 cases was attributed to GBS [1]. The Centers for Disease Control and Prevention (CDC) publishes an annual GBS surveillance report, which gathers all microbiology data from aggregate surveillance areas at normally sterile sites. Between 2010 and 2020, the number of cases of individuals older than 18 years in monitored hospitals increased from 2,081 to 2,958 patients [6,7].

## Conclusions

Spinal epidural abscesses are relatively uncommon and can be challenging to diagnose due to their nonspecific symptoms. The presence of GBS as an invasive pathogen causing SEAs highlights the importance of considering this diagnosis, particularly in high-risk patients. If left untreated, SEA can result in permanent neurologic deficits due to spinal cord compression and, in severe cases, even death. Timely intervention and a more systematic approach to the diagnosis and treatment of SEA are crucial for achieving favorable outcomes for the patient.
